# Adding Branched-Chain Amino Acids and Vitamin D to Whey Protein Is More Effective than Protein Alone in Preserving Fat Free Mass and Muscle Strength in the First Month after Sleeve Gastrectomy

**DOI:** 10.3390/nu16101448

**Published:** 2024-05-11

**Authors:** Luigi Schiavo, Biagio Santella, Barbara Paolini, Farnaz Rahimi, Emmanuele Giglio, Barbara Martinelli, Stefano Boschetti, Lilia Bertolani, Katia Gennai, Simone Arolfo, Maria Paola Bertani, Vincenzo Pilone

**Affiliations:** 1 Department of Medicine, Surgery and Dentistry “Scuola Medica Salernitana”, University of Salerno, 84081 Baronissi, Italy; bsantella@unisa.it; 2 NBFC—National Biodiversity Future Center, 90133 Palermo, Italy; 3 Department of Innovation, Experimentation and Clinical Research, Unit of Dietetics and Clinical Nutrition, Santa Maria Alle Scotte Hospital, University of Siena, 53100 Siena, Italy; barbara.paolini@ao-siena.toscana.it (B.P.); barbara.martinelli@ao-siena.toscana.it (B.M.); katia.gennai@ao-siena.toscana (K.G.); 4 Dietetic Unit, Città della Salute e della Scienza Hospital, 10126 Turin, Italy; frahimi@cittadellasalute.to.it (F.R.); sboschetti@cittadellasalute.to.it (S.B.); 5 Department of Bariatric Surgery, Clinical Institute “Beato Matteo”, 27029 Vigevano, Italy; giglio.emmanuele@gmail.com (E.G.); lilia.bertolani@yahoo.it (L.B.); bertani.mariapaola@hsr.it (M.P.B.); 6 General Surgery, Department of Surgical Sciences, University of Turin, 10126 Turin, Italy; simone.arolfo@unito.it; 7 Public Health Department, University of Naples Federico II, 80131 Naples, Italy; vincenzo.pilone@unina.it

**Keywords:** sleeve gastrectomy, body composition, muscle strengh, protein supplementation, vitamin D, branched-chain amino acids

## Abstract

Objectives: Sleeve gastrectomy (SG) is one of the most commonly performed weight loss (WL) bariatric procedures. The main goal of WL is reducing total body weight (TBW) and fat mass (FM). However, TBW loss is systematically accompanied by a decline in fat-free mass (FFM), predominantly in the first post-surgical month, despite protein supplementation. Branched-chain amino acids (BCAAs) and vitamin D seem to attenuate loss of FFM and, thus, reduce the decline in muscle strength (MS). However, data on the role of an integrated supplementation with whey protein plus BCAAs plus vitamin D (P+BCAAs+Vit.D) vs. protein alone on total weight loss (TWL), fat mass (FM), fat-free mass (FFM), and (MS) in the first month after SG are lacking. Therefore, the present study aims to evaluate the impact of P+BCAAs+Vit.D vs. protein alone supplementation on TWL, FM, FFM, and MS in the first month after SG. Materials and Methods: Before SG and at 1 month afterward, we prospectively measured and compared TBW, FM, FFM, and MS in 57 patients who received either a supplementation with P+BCAAs+Vit.D (n = 31) or protein alone (n = 26). The impact of P+BCAAs+Vit.D and protein alone supplementation on clinical status was also evaluated. Results: Despite non-significant variation in TBW, FM decreased more significantly (18.5% vs. 13.2%, *p* = 0.023) with the P+BCAA+Vit.D supplementation compared to protein alone. Furthermore, the P+BCAA+Vit.D group showed a significantly lower decrease in FFM (4.1% vs. 11.4%, *p* < 0.001) and MS (3.8% vs. 18.5%, *p* < 0.001) compared to the protein alone group. No significant alterations in clinical status were seen in either group. Conclusion: P+BCAA+Vit.D supplementation is more effective than protein alone in determining FM loss and is associated with a lower decrease in FFM and MS, without interfering with clinical status in patients 1 month after SG.

## 1. Introduction

Bariatric and metabolic surgery (BMS) has consistently demonstrated its safety, effectiveness, and long-term benefits in addressing severe to moderate obesity and associated comorbidities on a global scale [1]. Among the array of bariatric procedures, sleeve gastrectomy (SG) has emerged as one of the most prevalent surgical interventions [2]. It is widely acknowledged that a primary objective in weight management, even following BMS, is to optimize the reduction of fat mass (FM) while safeguarding metabolically active fat-free mass (FFM) [3,4,5]. However, it is observed that BMS precipitates substantial weight loss, encompassing reductions in both FM and FFM, with a notable decline in muscle mass evident within the initial month post-SG [6,7]. Notably, Maïmoun et al. highlight that the immediate and pronounced weight reduction one month post-SG (mean weight loss, −9.8 ± 2.6 kg) is not solely attributable to FM loss (−8.3 ± 4.0%), but also entails a discernible decline in FFM (ranging from −7.3% to 9.5%) [6]. Ensuring the preservation of adequate FFM assumes critical importance in formulating dietary recommendations for weight management, even post-BMS, given the pivotal role of muscles in overall protein metabolism [8]. Moreover, a significant reduction in FFM has been associated with adverse effects on the resting metabolic rate (RMR) [9], deceleration of weight loss rates, susceptibility to weight regain [10], and heightened risks of diminished muscle strength (MS) and sarcopenia [11,12]. Consequently, it is imperative to implement preemptive measures promptly following BMS to mitigate FFM loss and subsequently uphold MS. Nutritional guidelines currently advocate for an average daily protein intake post-BMS, typically ranging from 90 to 120 g or 1.1 g/kg of ideal body weight, to mitigate the undesired loss of FFM [13]. However, the effects of post-BMS protein supplementation remain poorly understood, with existing studies predominantly focusing on dietary protein intake [4,14,15,16]. Within this context, the quality of protein sources gains significance, particularly concerning the content of leucine, a branched-chain amino acid (BCAA), and vitamin D levels, both crucial for FFM maintenance [17]. Leucine plays a pivotal role in fostering anabolic effects by enhancing protein synthesis and reducing protein degradation, thereby promoting a positive net muscle protein balance [18]. Moreover, maintaining adequate leucine intake is vital for healthy muscle tissue, with research indicating its regulatory role in muscle protein synthesis and its influence on long-term body composition [19]. Studies comparing different protein beverages have indicated that higher leucine concentrations provide superior stimulation to muscle protein synthesis, resulting in reduced muscle catabolism [17]. Additionally, isoleucine and valine, the other two BCAAs, contribute significantly to preserving muscle mass hypertrophy [17]. Vitamin D, owing to its receptors in muscle tissue, plays a crucial role in muscle function [20]. Several clinical studies have highlighted the relationship between vitamin D levels and MS, as assessed by hand grip force, demonstrating that lower vitamin D levels correlate with greater losses in FFM and MS [21]. Furthermore, recent reviews have indicated that daily supplementation with at least 400 IU of vitamin D enhances skeletal muscle force by an average of 17% [22]. In summary, ensuring adequate daily intake of BCAAs and vitamin D, combined with whey protein supplementation, may be crucial for maintaining FFM levels and MS during weight loss induced by BMS. Moreover, despite the recognized importance of MS, limited research has investigated the impact of BMS on it. Contrary to conventional beliefs regarding FFM decrease post-BMS, there is a lack of data on the role of integrated supplementation with protein plus BCAAs plus vitamin D versus protein alone on total weight loss (TWL), FM, FFM, and MS in the initial month following SG. Therefore, this study aims to assess the clinical implications of such supplementation strategies in the first month post-SG.

## 2. Materials and Methods

### 2.1. Study Design and Patients’ Selection

A multicenter prospective cohort study was conducted on a cohort of 57 patients with obesity who received SG between January and July 2023 in four Italian bariatric centers (Salerno, Siena, Turin, and Vigevano). 

In agreement with the most recent interdisciplinary Italian guidelines on metabolic and bariatric surgery [23], inclusion criteria were a body mass index (BMI) ≥ 40 kg/m^2^ or ≥35 kg/m^2^ with obesity-related comorbidities and age between 18 and 65 years.

All procedures performed in this study were in accordance with the ethical standards of the institutional and/or national research committee and with the 1964 Declaration of Helsinki and its later amendments. We did not apply to the ethics committee for this study because we classified our protocol in the categories of “*negligible risk research*” (research in which there is no foreseeable risk of harm or discomfort). Furthermore, for the purpose of the study, the used formulation (Dixipro HP) is not experimental but is already register and included as “food for special medical purposes” in the register of the Italian Minister of Health (code number 944885767) and therefore already has all the authorizations to be prescribed to patients with obesity undergoing BMS.

The Dixypro HP supplement was kindly provided free of charge to all trial participants by Bioitalia srl, which had no role in designing the trial, or in patient enrollment in the different participating sites, or in processing any trial-related data. Informed written consent was obtained from each participant after being informed about the purpose and nature of the study.

### 2.2. Post-SG Diet and Supplementation

Before starting the post-SG diet, candidates were counseled individually about the diet and supplementation that they would be expected to follow for 4 weeks. After discharge, patients assumed a liquid diet that was changed to a puree-based diet after 10–15 days and, after 3–4 additional weeks, to a soft solid food diet. 

Consistent with current recommendations [24], diet composition was adjusted to provide 1.0 g/Kg/ideal body weight proteins. To ensure that all 57 patients that were included consumed a similar diet, we developed a single postoperative isocaloric diet that was used by all involved bariatric centers. 

**Figure 1:**
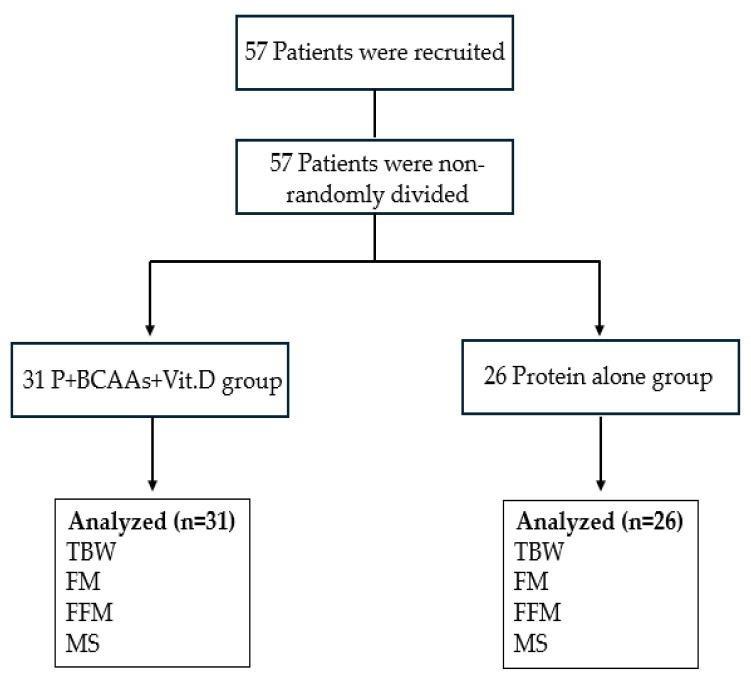
Flow chart comparing a 4-weeks post-operative supplementation with whey protein, 40 g; vitamin D, 2000 UI; L-leucine, 40 mg; L-isoleucine, 20 mg; L-valine, 20 mg [P+BCAA+Vit.D group] vs. protein alone group. TBW = total body weight; FM = fat mass; FFM = fat-free mass; MS = muscle strength.

Concerning post-SG supplementation, considering that patient’s personality, maturity, knowledge, and understanding represent the most critical barriers to and/or facilitators of adherence to prescribed supplementation after BMS [25], we decided to non-randomly allocate the patients into two groups, assigning them to the group that was judged most suitable for their specific circumstances and/or conditions (Figure 1): the P+BCAA+Vit.D group (n = 31) that followed a supplementation with whey protein, 40 g; vitamin D, 2000 UI; L-leucine, 40 mg; L-isoleucine, 20 mg; L-valine, 20 mg (Dyxypro HP, Bioitalia, Italy) and the protein alone group (n = 26), which followed a supplementation with whey protein, 40 g. 

During this follow-up period, patients were not encouraged to modify their physical activity. All patients were evaluated the day before the SG (baseline) and 1 month after the procedure. 

### 2.3. Anthropometric, Body Composition and MS Assessment of the Study Population

In all patients, body weight (kg) and height (cm) were determined under standard conditions (fasting state, light street clothes with shoes, and any other heavy items removed). Height was measured using a mechanical measuring tape. TBW was assessed using a bariatric digital scale. Body mass index (BMI) was calculated by dividing body weight (kg) by height (m^2^) [26]. 

Patients’ body compositions were measured by bioelectrical impedance assay (BIA). In all participating centers, the BIA instrument used was the latest generation in body composition analysis, using the latest multi-frequency technology, and it was in compliance with the requirements of Directive 90/384/EEC for weighing with non-automatic devices in the medical sector and Directive 93/42/EEC for medical devices. To perform an appropriate analysis, as we previously reported [26], all patients were required to comply with these conditions prior to the BIA: no food ingestion for at least 4 h, minimal intake of 2 L of water the day before, no physical activity for at least 8 h, no coffee or alcoholic beverage consumption for at least 12 h, and no diuretic use for at least 24 h. Patients were also asked to empty their bladder immediately prior to the BIA test. Concerning patient’s MS, a hand dynamometer was used to measure absolute hand grip strength. Strength was measured using the dominant hand. Each participant was in a seated position with the arm flexed at 90° and was asked to squeeze the dynamometer at maximum strength for 3 s, and they were allowed to rest for 15 s between measurements. The maximum effort expended (kg) was referred as absolute MS [27].

### 2.4. Statistical Analysis 

The effects of post-SG P+BCAA+Vit.D and protein alone programs were analyzed using a *t*-test for continuous variables. To compare variables within and between groups, a paired *t*-test and Student’s *t*-test were used, respectively. GraphPad Prism for Windows (version 9.1.2p) was used for statistics (Graph Pad Software, La Jolla, CA, USA). Data are reported as mean ± standard deviation (SD), and a *p*-value < 0.05 was considered statistically significant. Furthermore, any *p* value less than 0.0001 was conventionally stated as *p* < 0.001. 

## 3. Results

### 3.1. Preoperative Characteristics of the Study Group

The study included 57 patients (38 females and 19 males) with a mean age of 43 (±11.7) years. Before surgery, the P+BCAA+Vit.D and protein alone groups were comparable in terms of TBW (*p* = 0.711), BMI (0.710), FM (*p* = 0.564), FFM (*p* = 0.863), and MS (*p* = 0.623). 

### 3.2. Impact of P+BCAAs+Vit.D vs. Protein Alone on TBW, BMI, FM, FFM, and MS

No patients dropped out of the study. As expected, at follow-up (4 weeks post-SG), we observed a significant improvement in TBW and BMI in both groups studied (P+BCAAs+Vit.D: TBW and BMI *p* = 0.026 and *p* = 0.040, respectively; protein alone: TBW and BMI, *p* = 0.04 and *p* < 0.027, respectively) (Table 1). However, as shown in Table 1, we did not observe any significant difference between the two groups in terms of TBW (*p* = 0.994) and BMI lost (*p* = 0.401). 

Furthermore, as shown in Table 1, we observed a significant decrease in FM in both groups study (P+BCAAs+Vit.D: FM *p* < 0.001; protein alone: FM, *p* = 0.030). However, we observed a greater loss of FM in the P+BCAA+Vit.D group in comparison with protein alone group (18.5% vs. 13.2%, *p* = 0.023). Conversely, as shown in Table 1, we observed a significant decrease in FFM (*p* = 0.041) and MS (*p* = 0.047) in protein alone group, whereas we did not observe any significant changes in FFM (*p* = 0.485) and MS (*p* = 0.675) in the P+BCAA+Vit.D group. 

Moreover, as shown in Table 1, we observed a reduced loss of FFM in the P+BCAA+Vit.D group compared to the protein alone group (4.1% vs. 11.4%, *p* < 0.001). Similarly, we observed a reduced loss of MS in the P+BCAA+Vit.D group compared to the protein alone group (3.8% vs. 18.5%, *p* < 0.001) (Table 1). 

Additionally, as reported in Table 1, we observed a further advantage in combining P+BCAAs+Vit.D compared with protein alone on FFM and MS levels (*p* < 0.001).

### 3.3. Impact of P+BCAAs+Vit.D vs. Protein Alone on Patient’s Clinical Parameter

As reported in Table 2, we found a significant amelioration of general clinical status in both groups studied, including levels of liver enzymes, triglycerides, cholesterol, and creatine, while no changes were observed for markers of renal function.

## 4. Discussion

Our study’s main finding indicates that supplementation with P+BCAAs+Vit.D proves more effective than protein alone in promoting FM loss while mitigating the decrease in FFM among patients one month after SG. Notably, despite the well-established understanding that skeletal muscle mass diminishes during weight loss induced by dietary restriction, resulting in reduced MS [11,12,27], limited research has explored the impact of BMS on MS. Herein, we present, to the best of our knowledge, the first evidence suggesting that P+BCAAs+Vit.D, compared to protein alone, correlates with a lesser decline in MS among patients one month post-SG. Our study builds upon previous observations, particularly those highlighted by Maïmoun et al. and Jung et al., emphasizing that the rapid weight loss observed one month after SG is not solely attributed to reductions in FM but also entails significant loss of FFM [6,7]. Unfortunately, from a clinical perspective, the rapid decline in FFM may compromise metabolic rate, aerobic capacity, and overall functional ability, posing significant implications for individuals’ physical well-being, metabolic health, and overall health status [28]. Additionally, FFM plays a critical role in basal metabolic rate, body temperature regulation, maintenance of skeletal muscle integrity and strength, functional capacity, and overall quality of life [28]. Therefore, preserving FFM or minimizing its loss during FM reduction is deemed optimal and has been termed “high-quality weight loss” [29]. As highlighted by Rondanelli et al., adequate daily intake of whey protein proves beneficial in preserving FFM [17]. Consistent with this, our recent study demonstrated that a protein-enriched diet (2.0 g/kg of ideal body weight) post-BMS is more effective in promoting FM loss while preserving FFM compared to a normal-protein diet (1.0 g/kg of ideal body weight), without adverse effects on renal function in male patients post-BMS [4]. These findings align with previous research by Mettler et al., indicating that dietary protein consumption at 2.3 g/kg/day is superior to the recommended dietary allowance (RDA) of 1.0 g/kg/day for maintaining FFM [30], and with data by Pasiakos et al., suggesting that a daily protein intake of 1.6 g/kg/day (twice the RDA) is sufficient to protect FFM during short-term weight loss [31]. Furthermore, while BMS leads to diminished muscle mass with weight loss, the postoperative changes in MS remain poorly understood. Alba et al., in their examination of lean mass and MS changes following gastric bypass surgery, noted declines in lean mass and absolute MS amidst significant weight loss; notably, the majority of this decline occurred within the first six months post-operation [27]. These findings are consistent with other studies demonstrating reductions in lean mass and absolute MS in the early post-BMS period [32,33,34,35,36,37]. Nevertheless, our findings suggest that the addition of BCAAs and vitamin D, in conjunction with whey protein, could offer further strategies to mitigate the loss of MS as well as FFM. One potential rationale for this lies in the demonstrated ability of BCAA supplementation to prevent protein loss in conditions marked by muscle protein wasting [36]. At the same time, BCAA supplements tend to optimize muscle protein synthesis during energy deficit states, countering protein disarrangement and preserving energy homeostasis [38,39]. Specifically, BCAAs exhibit anabolic effects by boosting protein synthesis and decreasing protein degradation [40]. Moreover, BCAAs can interact with the insulin metabolic pathway, thereby modulating protein synthesis and maintaining FFM during caloric restriction. Given that BCAAs also influence glucose utilization by skeletal muscle, stimulating the glucose-alanine cycle and facilitating glucose reuse, adequate leucine supply is considered a potential strategy for managing obesity by preserving FFM and MS [41,42]. Consequently, our results strongly suggest that ensuring an adequate daily intake of BCAAs may play a pivotal role in maintaining FFM and MS during weight loss induced by a low-calorie diet in the first month post-BMS. Moreover, in clinical contexts, the significant relationship between vitamin D and muscle function has been underscored in various studies, indicating that blood vitamin D levels correlate with MS as assessed by handgrip strength, underscoring the necessity for adequate oral vitamin D supplementation to uphold FFM and MS [21,43,44,45]. Another possible rationale lies in the fact that vitamin D supplementation has been shown to increase calcium accumulation within the sarcoplasmic reticulum by enhancing the quantity of calcium-binding receptors and improving the efficacy of calcium-binding sites, as well as facilitating phosphate transport across cell membranes. Consequently, this process promotes the proliferation and differentiation of muscle cells [17]. We acknowledge certain methodological limitations in our study, notably the small sample size of patients and the uneven distribution of males and females (38 females in addition to 19 males). However, this gender disproportion reflects the higher prevalence of women undergoing surgical treatment for BMS in the participating departments. Additionally, our assessment of body composition relied solely on bioelectrical impedance analysis (BIA) without supplementary measures such as computed tomography. Nevertheless, BIA offers several advantages when compared to other approaches, including safety, non-invasiveness, affordability, ease of use, reproducibility, and integration into medical routines [46]. While isokinetic dynamometry is typically considered the gold standard for assessing strength [47], we opted for handgrip strength due to its simplicity, non-invasiveness, and high reproducibility in measuring absolute muscle strength [27]. Our study’s novelty lies in implementing an early postoperative strategy aimed at preserving FFM and MS through a combination of dietary adjustments and the use of a comprehensive formula supplement (DIXIPRO HP). This supplement stands out for its inclusion of high biological value proteins, which, when combined with an appropriate protein-enriched diet, may help preserve FFM and MS in obese patients. Furthermore, it contains BCAAs and Vitamin D, which are believed to play crucial roles in maintaining FFM and MS during weight loss induced by low-calorie diets, such as those followed in the initial month after BMS.

## 5. Conclusions

Based on our findings, we are to support the hypothesis that P+BCAA+Vit.D supplementation is more effective than protein alone in determining FM loss and is associated with a lower decrease in FFM and MS, without interfering with clinical status in patients 1 month after SG. These results should be confirmed in a larger randomized trial with longer follow-up periods and larger sample size.

## Figures and Tables

**Table 1:** Post-operative characteristics of the study groups.

Parameter	Group	Baseline	4-Weeks Follow-Up	*p* Value *	*p* Value **
Total body weight (kg)	P+BCAA+Vit.D	116.1 ± 22.5	103.6 ± 20.6	0.026	0.994
	Protein alone	118.4 ± 22.7	105.9 ± 20.1	0.040	
BMI (kg/m^2^)	P+BCAA+Vit.D	42.8 ± 5.98	38.3 ± 5.93	0.004	0.401
	Protein alone	43.3 ± 7.00	39.2 ± 5.82	0.027	
Fat Mass (kg)	P+BCAA+Vit.D	53.7 ± 11.1	43.8 ± 10.3	<0.001	0.023
	Protein alone	55.6 ± 12.4	48.2 ± 11.4	0.030	
Fat-Free Mass (kg)	P+BCAA+Vit.D	57.1 ± 13.6	54.7 ± 12.8	0.485	<0.001
	Protein alone	57.7 ± 12.2	51.1 ± 10.4	0.041	
Muscle Strenght (kg)	P+BCAA+Vit.D	39.1 ± 14.3	37.6 ± 13.4	0.675	<0.001
	Protein alone	38.6 ± 13.4	31.3 ± 12.4	0.047	

BMI = body mass index; The values are expressed as mean ± standard deviation (SD); * = 4 weeks follow-up vs. baseline; ** = 4 weeks follow-up P+BCAAs+Vit.D vs. protein alone.

**Table 2:** Patients’ clinical parameters at baseline and after 4 weeks.

Clinical Characteristics	Group	Baseline	4-Week Follow-Up	*p*
Glucose (mg/dL)	P+BCCAs+Vit. D	122.1 ± 60.37	91.6 ± 18.62	0.011
	Protein alone	108.6 ± 16.48	91.2 ± 13.18	<0.001
Insulin (mU/L)	P+BCCAs+Vit. D	25.9 ± 17.67	14.5 ± 8.32	<0.001
	Protein alone	28.7 ± 21.27	16.9 ± 10.83	0.015
HOMA Index	P+BCCAs+Vit. D	8.74 ± 9.83	3.31 ± 2.14	0.005
	Protein alone	7.80 ± 6.38	3.80 ± 2.42	0.005
Hemoglobin A1C (%)	P+BCCAs+Vit. D	6.04 ± 1.68	5.52 ± 1.10	0.155
	Protein alone	5.54 ± 0.75	5.00 ± 0.80	0.015
Creatine (mg/dL)	P+BCCAs+Vit. D	0.80 ± 0.19	0.85 ± 0.34	0.310
	Protein alone	0.78 ± 0.17	0.86 ± 0.25	0.157
GFR (mL/min)	P+BCCAs+Vit. D	102.1 ± 15.42	100 ± 15.85	0.611
	Protein alone	98.9 ± 12.42	95.5 ± 20.88	0.479
Iron (ng/dL)	P+BCCAs+Vit. D	62.5 ± 32.14	63.5 ± 23.39	0.886
	Protein alone	71.6 ± 17.02	67.1 ± 22.88	0.425
Uric Acid (mg/dL)	P+BCCAs+Vit. D	5.63 ± 1.41	6.03 ± 1.92	0.347
	Protein alone	5.68 ± 1.17	6.06 ± 2.28	0.397
Total cholesterol (mg/dL)	P+BCCAs+Vit. D	204.4 ± 46.28	170.1 ± 28.51	0.001
	Protein alone	185.4 ± 45.10	167.3 ± 39.37	0.129
HDL (mg/dL)	P+BCCAs+Vit. D	49.6 ± 11.37	44.6 ± 10.87	0.082
	Protein alone	52.50 ± 21.37	47.8 ± 13.65	0.351
Triglycerides (mg/dL)	P+BCCAs+Vit. D	158.7 ± 132.76	124.1 ± 68.19	0.204
	Protein alone	122.8 ± 69.90	114.7 ± 51.88	0.637
GOT (U/L)	P+BCCAs+Vit. D	24.5 ± 13.82	33.0 ± 18.21	0.043
	Protein alone	27.6 ± 23.87	31.0 ± 19.65	0.583
GPT (U/L)	P+BCCAs+Vit. D	30.2 ± 20.11	40.4 ± 23.68	0.073
	Protein alone	37.6 ± 37.24	41.4 ± 32.87	0.703
GGT (U/L)	P+BCCAs+Vit. D	33.4 ± 24.39	29.4 ± 16.89	0.456
	Protein alone	32.3 ± 20.10	30.4 ± 19.62	0.728
ESR (mm/h)	P+BCCAs+Vit. D	20.8 ± 11.92	19.3 ± 13.25	0.637
	Protein alone	17.4 ± 10.83	18.9 ± 12.65	0.631
RCP (mg/L)	P+BCCAs+Vit. D	3.62 ± 4.70	1.29 ± 1.92	0.015
	Protein alone	4.12 ± 8.24	1.51 ± 3.00	0.139
Na (mEq/L)	P+BCCAs+Vit. D	140.9 ± 3.31	141.3 ± 2.16	0.291
	Protein alone	141.2 ± 2.23	142.3 ± 2.65	0.119
K (mEq/L)	P+BCCAs+Vit. D	4.36 ± 0.41	4.14 ± 0.44	0.046
	Protein alone	4.37 ± 0.41	4.26 ± 0.47	0.368
Cl (mEq/L)	P+BCCAs+Vit. D	103.3 ± 3.08	103.4 ± 2.14	0.849
	Protein alone	104.1 ± 2.95	103.5 ± 2.52	0.422
Vitamin D (ng/mL)	P+BCCAs+Vit. D	24.3 ± 3.1	31.4 ± 2.14	0.031
	Protein alone	22.4 ± 2.75	23.2 ± 2.21	0.295

HOMA Index = homeostasis model assessment; GFR = glomerular filtration rate; HDL high-density lipoprotein; GOT = glutamate-oxalacetate transaminase; GPT = glutamate-pyruvate transaminase; GGT = gamma-glutamyltransferase; ERS = erytrocyte sedimentation rate; RCP = reactive C Protein. Data are reported as mean ± standard deviation.

## Data Availability

The data included in this manuscript are derived from the University database. We are not authorized to share the data with third party organizations. However, the corresponding author is available to provide any explanation to the Editor if requested.
